# Transfer of Minibeam Radiation Therapy into a cost-effective equipment for radiobiological studies: a proof of concept

**DOI:** 10.1038/s41598-017-17543-3

**Published:** 2017-12-11

**Authors:** Y. Prezado, M. Dos Santos, W. Gonzalez, G. Jouvion, C. Guardiola, S. Heinrich, D. Labiod, M. Juchaux, L. Jourdain, C. Sebrie, F. Pouzoulet

**Affiliations:** 1 Laboratoire d’Imagerie et Modélisation en Neurobiologie et Cancérologie (IMNC), Centre National de la Recherche Scientifique (CNRS), Universités Paris 11 and Paris 7, Campus d’Orsay, 91405 Orsay, France; 2 Histopathologie Humaine et Modèles Animaux, Institut Pasteur, 28 Rue du Docteur Roux, 75015 Paris, France; 3 Institut Curie, PSL Research University, Translational Research Department, Experimental Radiotherapy Platform, Orsay, France; 4 Paris Sud University, Paris -Saclay University, 91405 Orsay, France; 5 Imagerie par Résonance Magnétique Médicale et Multi-modalités (IR4M-UMR8081), Université Paris Sud, 91405 Orsay, France

## Abstract

Minibeam radiation therapy (MBRT) is an innovative synchrotron radiotherapy technique able to shift the normal tissue complication probability curves to significantly higher doses. However, its exploration was hindered due to the limited and expensive beamtime at synchrotrons. The aim of this work was to develop a cost-effective equipment to perform systematic radiobiological studies in view of MBRT. Tumor control for various tumor entities will be addressable as well as studies to unravel the distinct biological mechanisms involved in normal and tumor tissues responses when applying MBRT. With that aim, a series of modifications of a small animal irradiator were performed to make it suitable for MBRT experiments. In addition, the brains of two groups of rats were irradiated. Half of the animals received a standard irradiation, the other half, MBRT. The animals were followed-up for 6.5 months. Substantial brain damage was observed in the group receiving standard RT, in contrast to the MBRT group, where no significant lesions were observed. This work proves the feasibility of the transfer of MBRT outside synchrotron sources towards a small animal irradiator.

## Introduction

The use of the spatial fractionation of the dose is a strategy to overcome the main limitation in radiotherapy (RT), namely the normal tissue tolerances. One example is Grid therapy^1,2^. Current clinical data confirm the value of Grid therapy in the palliative management of large volume of disease with an acceptable toxicity profile^1,2^. Grid patterns (along the two directions perpendicular to the beam) are typically delivered by conventional medical linear accelerators using beam sizes of around 1–2 cm^2^. Grid therapy faces some major limitations due to the important lateral scattering of megavoltage X-rays beam employed. This leads to a reduction in the peak-to-valley dose ratios (PVDR), an important indicator for normal tissue sparing^3^. Another drawback is the need of using “large” beam sizes (around one squared centimeter) not to dramatically decrease the dose rate.

The use of very narrow beams, as in synchrotron microbeam (MRT) and minibeam radiation therapies (MBRT), allows exploiting the dose–volume effect^4^: the smaller the beam size is, the higher the tolerances of the healthy tissue. The very first animal studies with synchrotron-generated x-ray microbeams (MRT) showed the remarkable tissue sparing effect of very narrow beams^5^. In these techniques the irradiation is performed with planar beams. The dimensions perpendicular to the beam direction are submillimetric in one direction, covering up to several centimeters in the other one^5,6^. In the case of MRT, the beam width ranges from 25 to 100 μm (interbeam distance of 200–400 μm), while in the case of MBRT, the beam widths are around 500–700 μm (interbeam distance being the double of the width). MRT and MBRT are typically delivered by synchrotron beams consisting in very intense kilovoltage x-rays. Remarkably high normal tissue dose tolerances (≥100 Gy in one fraction) have been observed in numerous biological experiments^5–9^. Significant tumour growth delay in aggressive animal tumour models was also observed^3,10–12^.

These outcomes seem to be the result of the participation of different biological mechanisms (not well understood yet) different from those in standard RT. In addition to so-called dose-volume effects^4^, there are indications that some other contributors, such as cohort effects^13,14^ and prompt vascular repair^13,15^, may be participating. Systematic in-depth evaluations are needed. However, the confinement of MRT and MBRT to synchrotrons has limited its worldwide exploration and comprehensive evaluations. The thicker beams used in MBRT makes that the dose profiles are not as vulnerable as those of MRT to beam smearing from cardiac pulsations^16^, therefore very high dose rates are not needed. In addition, the implementation is technically easier than in MRT. This motivated this evaluation of the feasibility transfer of MBRT into a small animal irradiator. The reduced costs and the wide availability of this type of irradiators would enable systematic radiobiological studies first, and provide a direct path towards clinical trials, in a later stage.

Along this line, we have modified a commercial small animal irradiator to make it suitable for MBRT irradiations. The main challenges to be faced were to obtain minibeam patterns with peak-to-valley dose ratio (PVDR) values similar to those at synchrotrons along with dose rates high enough to be able to deliver high doses (>50 Gy) in one fraction in a time compatible with rodent’s anaesthesia. There have been some attempts to do this transfer with thinner beams (microbeam radiation therapy (MRT)). Hadsell *et al*.^17^ employed carbon nanotubes to produce 300 μm wide beams with a dose rate of 21.7 mGy/s. Despite promising results, this technology is not widespread yet. The lower dose rates compared to those available at synchrotrons^18^ might lead to beam blurring of such thin beams due to cardiosynchronous pulsations^16^. In a recent work^19^ a conventional x-ray tube was employed to implement microbeam radiation therapy. However, neither the energy spectrum nor the dose rates (300 mGy/s) were compatible with the use of very narrow beams in MRT *in vivo* experiments due to the possible beam blurring.

This manuscript describes our strategy and shows its feasibility by means of a first *in vivo* experiment. Since our approach is especially suitable for tumours for which pulmonary and/or cardiac cycles have minimal effects, in particular, for presently difficult-to-treat neurological indications, normal rat brains were irradiated. Our main target are high-grade gliomas, that are the most aggressive and common brain tumors^20^. We aimed to assess the gain in brain tissue sparing via MBRT compared with conventional irradiation. Late radiation injury is the major, dose-limiting complication of brain irradiation. Inflammation, blood barrier disruption, vascular lesions, demyelination, radio-necrosis and edema are examples of injuries induced by the radiation^21^. The precise mechanisms underlying different types of radiation-induced central nervous system (CNS) lesions are not completely understood. Vascular involvement leads to ischemia, infarction, and necrosis^22^. The blood–brain barrier might also have a role in the pathophysiology of radiation-induced damage as maintenance of CNS barriers is essential for brain homoeostasis. Brain irradiation may also cause neuroinflammation, evidenced by the activation of microglia. After insult to the brain, astrocytes may also become activated in response to signals released by injured neurons or activated microglia^23^. Along this line, the irradiated animals were followed for 6.5 months to evaluate long-term effects. Areas of infarction, BBB breakdown and edema were assessed via signal abnormalities in MRI images. Histology analysis allowed the evaluation of tissue integrity, inflammation, necrosis and calcifications, among others.

## Materials and Methods

### Implementation of MBRT in a small animal irradiator

The Small Animal Radiation Research Platform (SARRP, XSTRAHL Ltd., UK)^24,25^ available at the Experimental Radiotherapy Platform of Curie Institute in Orsay^26^ was used. A tension of 220 kV and an intensity of 13 mA were employed with inherent and additional filtrations of 0.8 mm and 0.15 mm of Beryllium and Copper, respectively. This results in an energy spectrum with an effective energy of 69 keV. See supplemental material. The manufacturer reported a beam divergence of 20 degrees. The dose rates at the isocentre (*i*.*e*. 35 cm far away from the source) are 2.2 Gy/min and 1.0 Gy/min at 1 cm-depth in water for 1 cm^2^ and 0.5 mm^2^ field sizes, respectively. The dose rates were measured with gafchromic films.

**Figure 1:**
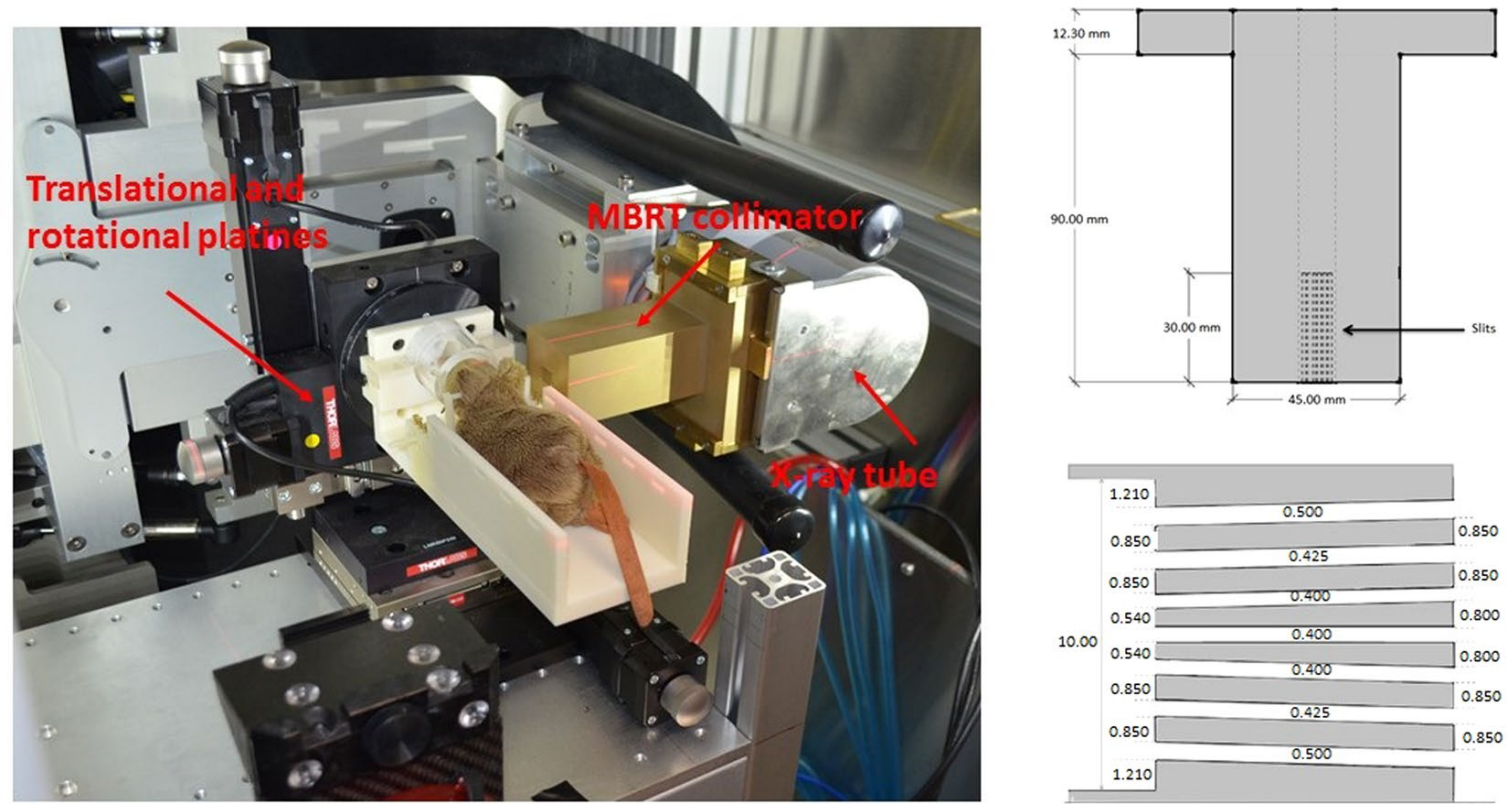
A photograph of the setup for MBRT rodents irradiation is show on the left. A longitudinal cross section of the minibeam collimator is depicted on the right (upper row). A zoomed cross section of the multislit collimator including the widths of the slits (white spaces) is shown on the right lower corner. All dimensions are given in mm.

A series of modifications of the SARRP were performed to make it suitable for MBRT experiments. First, a set of translation and rotation stages were placed on top of a custom made aluminium bench. The system reaches a precision of 1 μm and 5 arcmin allowing interlaced or cross-fired irradiations^12,27^. See Fig. 1. The shorter source-target distance (20 cm) provides an increase of dose rate a factor 3, approximately. The design of an adequate minibeam collimator was guided by means of Monte Carlo (MC) simulations (Geant4 toolkit version 10.01^28^). The Livermore electromagnetic physics list was employed. An energy cut of 250 eV was considered for all particles. The dose was scored in voxels of 50 μm × 5 mm × 1 mm. A global uncertainty of 4% was reached.

A divergent brass collimator was considered to compensate the large divergence (20 degrees). The widths of the slits were progressively increased from the centre towards the edges to homogenize the peak doses. The distances of the collimator from both the source and the target were also optimized. Peak to valley dose ratio (PVDR) values and full width half at maximum (FWHM) similar to those obtained at the European synchrotron radiation facility (ESRF)^18^ were used as figure of merit. In addition, centre-to-centre (ctc) distances doubling the FWHM (to get a good interlacing) were aimed for. A total maximum irradiation area of 1.2 × 2 cm^2^ was considered since the main goal of this first phase was to perform rat brain irradiation studies. A second collimator providing thicker (millimetre) beams was also manufactured. The dose distributions in a solid water phantom (20 cm × 20 cm × 4 cm) were calculated and were benchmarked against experimental data. The experimental dosimetry was performed following the two-steps protocol developed for synchrotron MBRT^18^.

### *In vivo* experiments

#### Ethics statement

All animal experiments were conducted in accordance with the animal welfare and ethical guidelines of our institutions. They were approved by the Ethics Committee of the Institut Curie and French Ministry of Research (permit no. 6361-201608101234488). Rats were anaesthetised with isoflurane (2.5% in air) during irradiation and magnetic resonance imaging (MRI). At the end of the study, the rats were terminally anaesthetised for brain fixation by the intracardiac perfusion of formalin zinc.

#### Irradiations

Two groups of animals were irradiated. A first group (series 1, n = 7) received conventional (broad beam) irradiations. The target was placed at the isocentre (*i*.*e*. 35 cm from the x-ray source) of the SARRP^25^. The rats brain, excluding the olfactory bulb and part of the cerebellum, was irradiated unilaterally. The irradiation field size at 1 cm-depth had a full width half at maximum of 1.1 cm. The dose rate was 2.2 Gy/min. A second series (n = 7) was irradiated with MBRT using the setup described in section 2.1 (series 2). Thus, the irradiation was not performed at the isocentre, but at a shorter distance to the source (20 cm). The total area covered was 1.2 × 1.2 cm^2^, including 7 minibeams. The dose rate was 3.5 Gy/min. A control group (n = 7) was also included in the study. The doses were calculated by using the treatment planning system of SARPP (Muriplan^29^) in the case of standard irradiation and employing Monte Carlo simulations for MBRT irradiations. In both cases, the calculations were performed in rats computed tomography images, therefore, the attenuation of cranial bone was considered. The same average dose at the centre of the rat brain was deposited in both series, 20 ± 2 Gy, which corresponds to 58 Gy peak dose in the MBRT case, as measured at 1 cm-depth in a solid water phantom. As explained in the introduction, our main target are gliomas. Radiation doses higher than 20 Gy are reported to be needed to obtain long-term survivals in glioma-bearing rats experiments^30^ after conventional irradiations. Therefore, we have chosen 20 Gy to perform a first evaluation on whether MBRT offers an advantage over conventional irradiation at doses high enough to reach a significant probability of glioma sterilization.

#### Follow up

The animals were followed-up for 6.5 months. The health status of each rat was checked once per week. The evaluation included weighting of the animals and the observation of the appearance of possible signs of distress. Those include lack of grooming, hyperreactivity, apathy, spontaneous sound vocalizations, troubles of movement among others. In case of sustained weight loss (>20% of the animal maximal weight), ataxia, prostration, troubles of movement, seizures or periorbital haemorrhages, the animals would be humanly killed.

An anatomical MRI study was performed at 6.5 months after irradiation in 3 out the seven animals in each series. For each imaging session, a catheter was inserted into the tail vein for contrast agent administration.

A 7-Tesla preclinical magnet (Bruker Avance Horizontal 7-T Bruker, Inc., Billerica, MA) equipped with a 35-mm-diameter “bird-cage” antenna was employed. Three series were acquired:

(i) Morphological T2-weighted (T2W) images with a repetition time (TR) of 2500 ms and 4 echo times (TE) of 11, 33, 55 and 77 ms. A signal averaging of 2 was employed. In all, 21 slides were acquired in a total time of 10 min 40 s.
(ii) T1-weighted (T1W) TurboRare sequences with a TR of 800 ms and a TE of 6.05 m. A signal averaging of 4 was employed. A total of 21 slides were acquired in a time of 5 min 7 s. Four acquisitions were performed, one before and three (5, 10 and 15 min) after the intravenous injection of a bolus of 100 µmol/kg Gd-DOTA (Guerbet SA, Villepinte, France).
(iii) T1 fast low-angle shot (FLASH) sequences with a TR and TE of 114.89 and 3.1 ms, respectively. A flip angle of 30° and a signal averaging of 4 were used. A total of 9 slides were acquired in a total time of 1 min 28 s. Acquisitions were made just before and 5, 10, and 15 min after the intravenous injection of a bolus of 100 µmol/kg Gd-DOTA (Guerbet SA, Villepinte, France).

In all sequences, the field of view was 35 mm × 35 mm, the in-plane resolution amounted to 0.14 mm × 0.14 mm, and the slice thickness and gap were 0.8 and 0.3 mm, respectively.

The main information provided by T2W sequences is the presence of increased fluid in diseased tissue that results in high signal intensity. One example is edema. The appearance of hematomas varies according to their age. The presence of high or dark signal in parallel in T1W helps disentangling the type of lesion. The observation of Gd leakage in the T1W images evidences a blood brain barrier (BBB) disruption. FLASH sequences, being very sensitive to Gd contrast and to methemoglobin products, help observing subtle BBB breakdowns, on the one hand, and distinguishing between different hemorrhagic processes, on the other.

At the end of the study, the rats were terminally anaesthetised for brain fixation by the intracardiac perfusion of a fixative solution (formalin zinc). The brains were then removed, fixed in the fixative solution, and embedded in paraffin; 4-μm-thick sections were cut and stained in haematoxylin and eosin (HE) for the histopathological (double-blinded) analysis was carried out by ECVP (European College of Veterinary Pathologists) board certified pathologist. Immunohistochemistry analysis was performed to assess the networks and cell morphologies of microglia (anti-Iba-1 antibody, Wako Chemicals, dilution: 1:500) and astrocytes (anti-GFAP antibody, Sigma-Aldrich, dilution: 1:500). This is a relevant information since astrocyte and microglial cell morphology is directly linked to their physiological state^31,32^. Contrary to HE staining, the specific immunohistochemistry analyses performed (Iba1 for microglial cells and GFAP for astrocytes) allow detection of cell processes, i.e. visualization of the cell organization in space. Neuroinflammation is characterized by an activation of microglial cells: proliferation, thickening and shortening of the cell processes. Destruction of the neuropil and nervous tissue leads to an activation of astrocytes: proliferation, increased number and thickening of their cell body and processes.

## Results

### Dosimetry features

**Figure 2:**
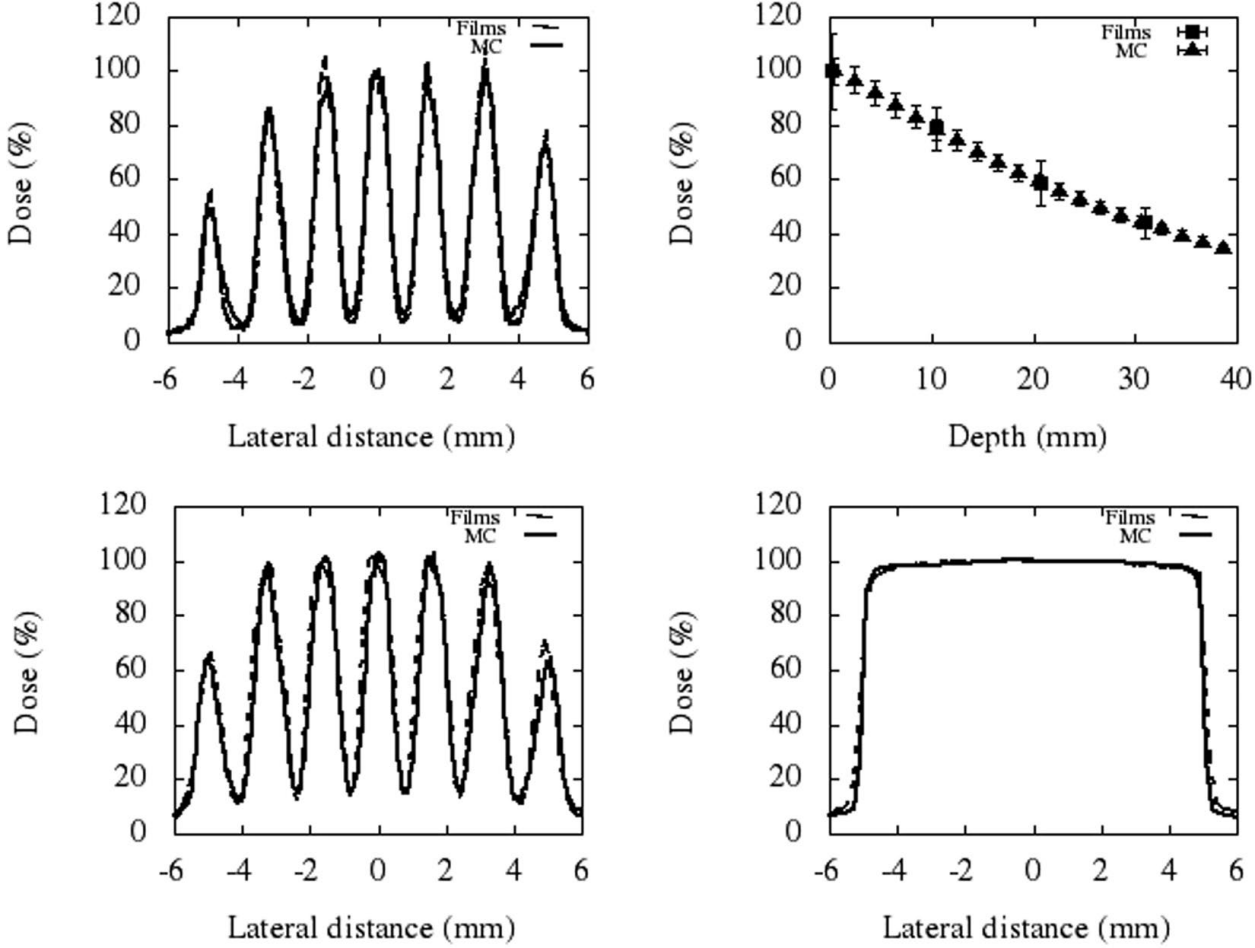
Comparison of experimental and calculated lateral dose profiles. The upper row shows the lateral dose profiles at 1 cm-depth (left) and the depth dose curve (right) for the central minibeam of the thinner collimator (FWHM of the central peak is 696 ± 20 μm at 1 cm-depth). The lower row depicts the lateral profiles at 1 cm-depth resulting after irradiation with the wider MBRT collimator (FWHM of the central peak is 968 ± 77 μm) and with the fixed SARPP collimator (irradiation at the isocentre).

Figure 1 depicts the geometry and dimensions of the designed collimator for MBRT. Figure 2 shows the lateral dose profile at 1 cm-depth (left) and the depth dose curve (right) for the central minibeam of the array obtained with that collimator. A good agreement is obtained between MC and experimental data. The criteria of acceptability for the dose calculations compiled in the Technical Report Series 430 (TRS 430) of the International Atomic Energy Agency was used^33^. The confidence limit (Δ) defined by Venselaar *et al*.^34^ was employed. See equation 1.

1 $${\rm{\Delta }}=|average\,deviation|+1.5\,SD$$

SD refers to standard deviation. Δ fits within the range of tolerances recommended by the TRS 430. Deviations smaller than 2% are obtained in the depth dose curves. A maximum Δ value of 3.6% is found in the high dose/small gradient regions of the profiles. Deviations smaller than 30% are found in the regions of low dose and small dose gradient of the profiles.

The FWHM of the central peak is 618 ± 18 μm and 696 ± 20 μm at the phantom surface and at 1 cm-depth, respectively. These values are similar to the ones obtained at the ESRF (640 μm)^3^.

Table 1 reports the comparison of PVDR values as a function of depth for synchrotron MBRT^3^ and this new approach for the collimator described above. The PVDR are higher with this new approach than in synchrotron MBRT in the first centimetre due to the use of a less energy spectrum. The decrease of PVDR in depth is more pronounced due to a much important divergence (20 degrees in the SARPP with respect to a negligible divergence at synchrotrons). Nonetheless, the PVDR are equal within the uncertainty bars for both synchrotron and our method, from 1 cm-depth.

**Table 1:** Comparison of PVDR values as a function of depth.

Depth (cm)	Synchrotron MBRT^3^	This work
0	15.2 ± 1.6	27.8 ± 2.8
1	9.6 ± 1.0	12.4 ± 2.3
2	9.3 ± 1.0	9.4 ± 2.0
4	8.6 ± 0.8	7.0 ± 1.8

The measured dose rate (central minibeam) amounted 3.5 ± 0.2 Gy/min at 1 cm depth. This allows depositing 50 Gy in 13 minutes, well within the time that rodents can stay under gaseous anaesthesia.

Therefore, the dosimetric features of our system are similar to the synchrotron ones^3^, excepting the dose rates (around 5000 Gy/s at the ESRF^12^, depending on working mode).

A second collimator with wider slits was also manufactured in order to evaluate the tolerance of normal (brain) tissue to thicker (millimetre) beams and it was used for the *in vivo* experiment presented in this work. The FWHM, ctc and PVDR at 1 cm depth (centre of rat brain) were 968 ± 77 μm, 1610 ± 128 μm and 6.8 ± 0.7, respectively. Figure 2 shows the lateral dose profile at 1 cm-depth (left) for this latter collimator, as well as that of the fixed SARRP collimator (right), both used for the *in vivo* experiment. The measured dose rate amounted 3.8 ± 0.2 Gy/min and 2.2 ± 0.1 at 1 cm depth for the MBRT and broad beam collimators, respectively.

### First *in vivo* experiment

All rats gained weight; no external evidence of brain damage was observed. The animals in series 1 (broad beam) presented moist desquamation starting between 2–3 weeks after irradiation. This was treated with Ialuset® PLUS (Laboratories Genevier). Permanent epilation was developed in all rats of series 1. In series 2 (MBRT), small skin lesions (desquamation) along with reversible epilation in the minibeam paths were observed.

**Figure 3:**
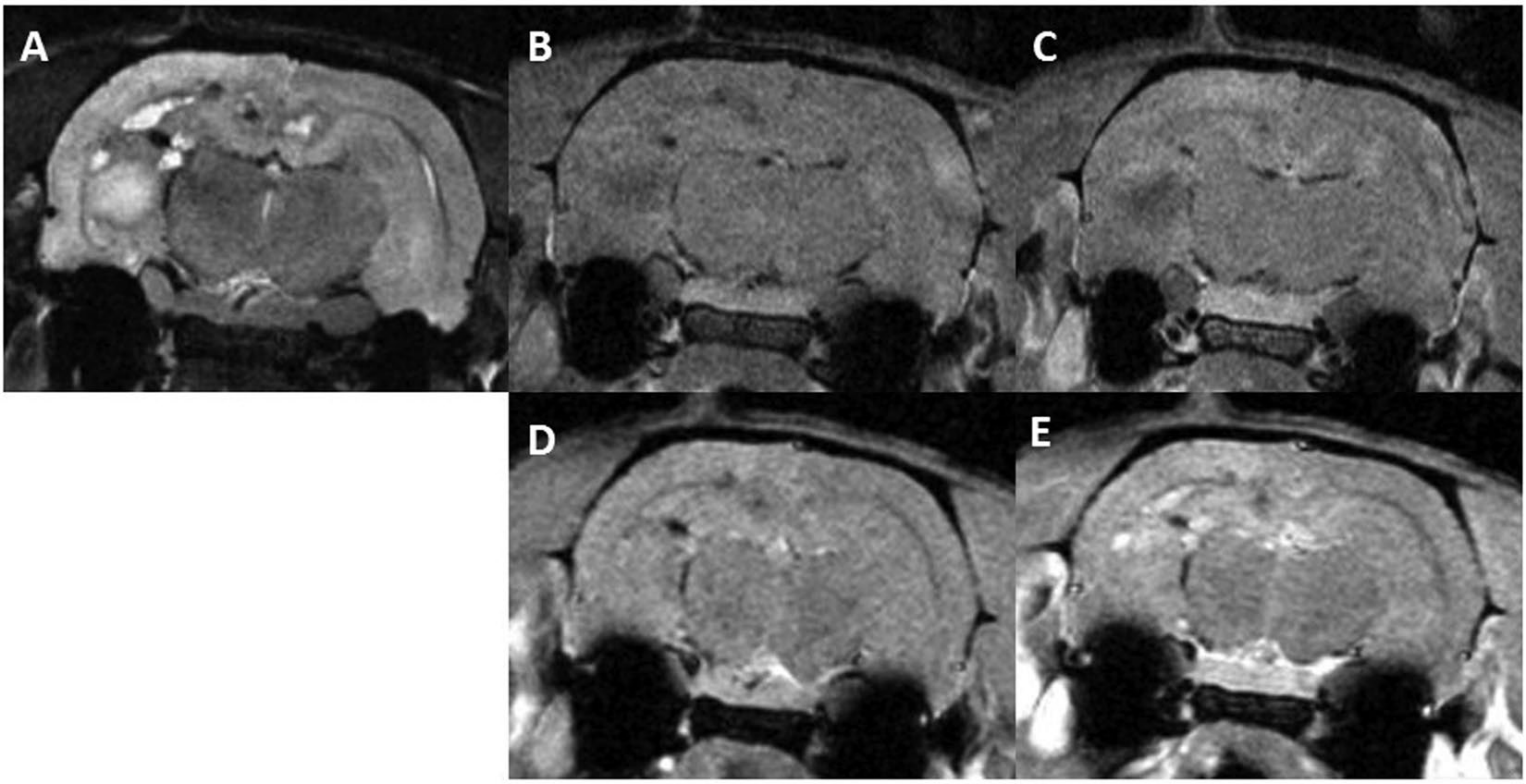
Axial MRI images of one rat in series 1: before Gd injection T2w (**A**), T1w (**B**) and T1 FLASH (**C**), T1w (**D**) and T1 FLASH after Gd injection (**E**). Substantial damage is observed. A large lesion with a high signal in T2w along with low signal in T1w and no BBB, compatible with an edema, is present in the left hippocampus. Several areas of chronic haemorrhage in the corpus callosum, subiculum and hippocampi presenting a rim of low intensity and a centre of high signal in T2w with an isotense signal in T1w and high intensity in T1w FLASH are observed. Some areas of bleeds are also present with low intensity both in T2w and T1w. An extensive BBB breakdown is also observed.

**Figure 4:**
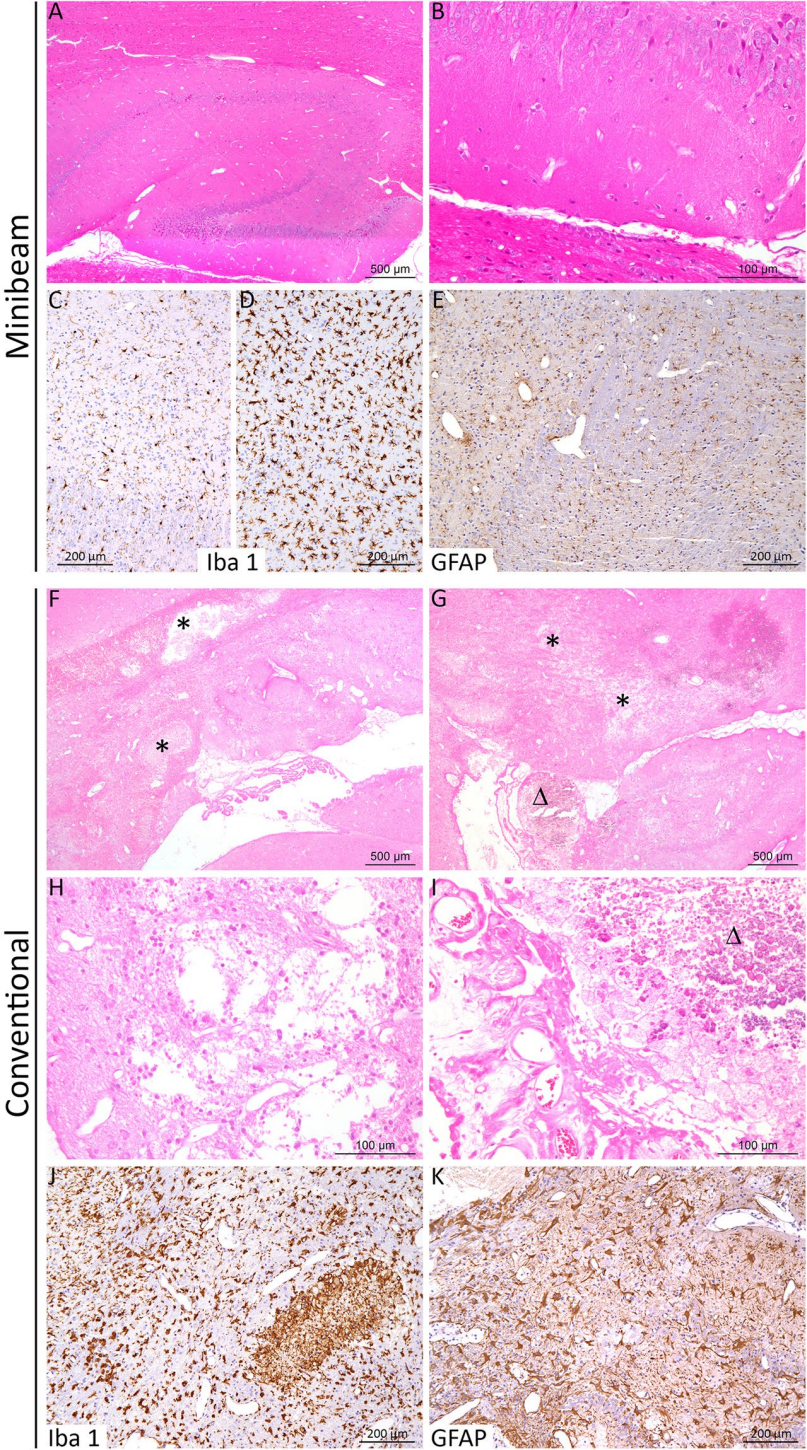
Histopathological lesion profile after conventional irradiation in contrast to minibeam RT. After minibeam irradiation (**A**–**E**), almost no significant histological lesion was detected (**A**,**B**). Immunohistochemistry analyses to specifically detect microglial cells (Iba-1) and astrocytes (GFAP) were carried out and revealed normal microglial cells in general (**C**), with microscopic foci of activation (neuroinflammation (**D**)) and a normal organization of the astrocyte network (**E**). After conventional irradiation (**F**–**K**), almost all rats displayed marked cerebral lesions (often located close to the hippocampus), characterized by: (**F**,**G**) edema, dilatation of ventricles, destruction of the brain parenchyma (necrosis) and cavitation (black star; higher magnification in (**H**)), associated with foci of calcification in the cortex or choroid plexus (**D**; higher magnification in (**I**)). In these lesions, a severe activation of microglial cells (**J**) and astrocytes (**K**) was also detected. (**A**,**B**,**F**–**J**)*: HE Staining;* (**C**,**D**,**J**): *Iba1 IHC;* (**E**,**K**): *GFAP IHC*.

Series 1 developed very important damage, particularly concentrated in hippocampus, subiculum, corpus callosum and part of the cortex. MRI images show large lesions compatible with hematomas and edema along with extensive blood brain permeability (BBB) in those areas. See Fig. 3. In addition, large ventricles, indicating cerebrospinal fluid accumulation, are observed in 2 out of the 3 animals imaged. The histopathological analysis (n = 7) also reveals severe lesions mostly concentrated close to the hippocampus: loss of nervous tissue, edema, vascular damage as well as neuroinflammation. The latter is characterized by an activation of microglial cells: proliferation, thickening and shortening of the cell processes. Destruction of the neuropil and nervous tissue leads to an activation of astrocytes: proliferation, increased number and thickening of their cell body and processes. See Fig. 4. Table 2 summarizes the findings for series 1. Important damage was observed in 6 out of the 7 rats evaluated.

**Table 2:** Summary of lesions observed in series 1.

MRI (n = 3)	• Edemas and/or haemorrhage in hippocampi, subiculum and corpus callosum (3/3)
	• Edemas and/or haemorrhage in cortex (2/3)
	• Lesions in pretectal region (2/3)
	• Lesions in thalamus (1/3)
	• Extensive BBB breakdown (3/3)
	• Large ventricles (2/3)
Histology (n = 7)	• Large necrosis (4/7)
	• Necrosis with cavitation (2/7)
	• Edemas (4/7)
	• Calcifications (1/7)
	• Microglial and astrocyte activation (6/7)
	• No lesions (1/7)

**Figure 5:**
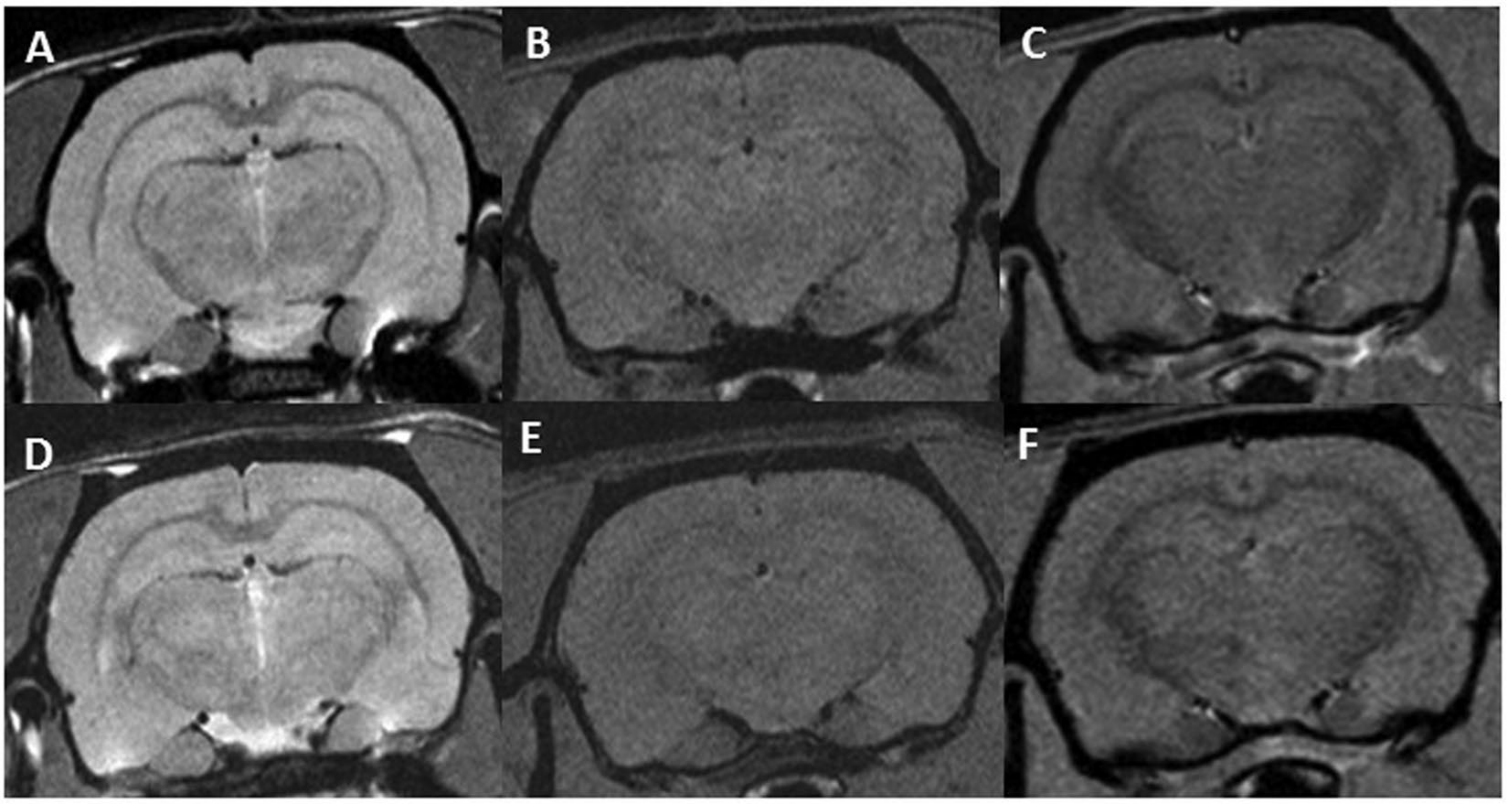
Axial MRI images of two rats. The upper row show the T2W, T1W and T1 FLASH (this later after Gd injection) images (**A**–**C**, respectively) for a rat receiving MBRT. The lower row depicts the same sequences for one rat belonging to the control group. No significant differences between the two groups are observed.

Concerning series 2, no significant lesions were observed in the MRI follow up. See Fig. 5. No substantial damage was found in the histological evaluation. Immunohistochemistry analyses revealed normal microglial cells in general, with some microscopic foci of activation (neuroinflammation) and a normal organization of the astrocyte network. See Fig. 4.

## Discusion and Conclusions

The treatment of radioresistant tumours, such as gliomas, remains one of the major challenges in RT. This limitation is especially severe in children, due to the high risk of complications in the development of the central nervous system. The management of tumours close to an organ of risk, like the spinal cord, is also restrained. Finding novel approaches that allow increasing normal tissue resistance is of utmost importance. This would make it possible to escalate tumour dose, resulting in an improvement in cure rate.

This is the motivation for the development of novel RT approaches based on distinct dose delivery methods like, synchrotron MRT and MBRT. The dose delivery methods employed in MBRT constitute a rupture with standard RT. MBRT has been shown to significantly increase the normal tissue resistance in animal experiments^6,8,9^ with respect to standard RT. Due to the need of intense low energy x-rays beams, MBRT along with other synchrotron RT techniques, like microbeam RT^5^, have been restricted to very large synchrotrons. This confinement has very much limited the worldwide exploration of this approach. The main goal of the present study was to evaluate the implementation of MBRT into inexpensive and widespread equipment. This would enable the realization of comprehensive radiobiological experiments to assess the (potential) increase of therapeutic index for various tumour sites as well as to unravel the biological mechanisms involved. With that aim, we have introduced a series of modifications in a SARRP system, including a suitable collimator, enabling to obtain similar dosimetric features (beam widths, PVDR) than those ones at synchrotrons as well as dose rates compatible with MBRT rodents irradiations. These measurements demonstrate the feasibility of such an implementation.

A first *in vivo* experiment was then performed in the modified system. The goals were twofold: firstly, to show the feasibility of performing MBRT irradiations in rodents in our system, and, secondly, to assess whether a gain in neurotoxicity reduction is still attained when thicker beams than the ones used at synchrotrons (500–700 μm) are employed. With that aim, we have compared the side effects of a classical homogenous RT and MBRT irradiations in an *in vivo* animal model (rat brain). The same (high) average dose (20 Gy in one fraction) was delivered to both groups, covering the almost totality of the brains (excluding olfactory bulb). Since late radiation injury is the major, dose-limiting complication of brain irradiation, the animals were followed up for 6.5 months. The animals receiving standard RT developed important skin and very severe long-term brain damage, including radionecrosis. In contrast, less severe skin damage and almost no brain damage was observed in the MBRT group. Only some microscopic foci of microglial activation were detected (neuroinflammation). It should also be emphasized that this gain in tissue resistance has been obtained millimetre beams (968 ± 77 μm at 1-cm depth) and low PVDR values (6.8 ± 0.7). It is a valuable finding in the context of general indication in the literature that the minibeams’ tissue-sparing effect starts to rapidly decline above 0.7 mm beam thickness when used with much higher x-ray minibeam doses^6^. To the best of our knowledge this is the first time that such an evaluation is done with millimetre-sized minibeams in the brain. The gain in tissue sparing with thick (mm-sized) beams have important implications for potential clinical trials, since it opens the door for future implementations with less technically demanding and very compact systems and, even with modified Linear Medical Accelerators. This would allow a rapid and direct route to proceed to clinical trials first, and to be able to reach a large number of patients, in a later stage.

The large divergence of these systems leads to a widening of the beams as a function of depth, resulting in a reduction of PVDR in depth. A (quasi) homogenisation of the dose starts from 8 cm-depth for the range of energies, beam widths and ctc distances used in this study, as assessed by Monte Carlo simulations. A similar behaviour is observed proton MBRT^35^, as a consequence of the multiple coulomb scattering. Although a reduced divergence is desirable, the aforementioned feature might still profit the treatment of deep seated brain tumors (>5 cm depth). The PVDR values in the first 5 centimetres are similar to those ones encountered in previous studies in X-rays and proton MBRT, leading to remarkable tissue tolerances^9,36^. The PVDR at 5 cm-depth gets down to 2.7 ± 0.1, but still comparable to those of proton MBRT^37^ and Grid therapy^38^. A tumor located in the centre of a human brain (around 8 cm-depth) would receive a quasi-homogeneous dose distribution. The use of different ctc distances would permit to adapt the PVDR values to the specific case. Irradiating from several directions, or the use of the use of interlaced or cross-fired arrays^12,27^ would serve to increase the dose in the tumor and to reduce the deposition in normal tissues. The availability of the system proposed here would permit the realization of biological experiments to evaluate the gain of the different configurations.

Despite their short penetration depth, the use of kilovoltage X-rays has already been successfully employed in some techniques like stereotactic synchrotron radiation therapy for brain tumor treatments^39^. Patients treatments would, nevertheless, benefit from higher beam energies. The optimum beam energy for MBRT from a dosimetric point of view was estimated to be 375 keV^40^.

Although the main goal of this work was to adapt a commercial system to perform systematic radiobiological studies in MBRT, the proof of concept established here could trigger further developments in this domain.

## Electronic supplementary material

Supplementary Information
