# Supplementary material for: Transfer of Minibeam Radiation Therapy into a cost-effective equipment for radiobiological studies: a proof of concept
Source: Sci Rep. 2017 Dec 11;7:17295. doi: 10.1038/s41598-017-17543-3 (PMC5725561; doi:10.1038/s41598-017-17543-3)

Transfer of Minibeam Radiation Therapy into a cost-effective equipment for radiobiological studies: a proof of concept

Y. Prezado[1*] and M. Dos Santos[1*], W. Gonzalez[1], G. Jouvion[2], C. Guardiola[1], S. Heinrich[3,4], D. Labiod[3,4], M. Juchaux[1], L. Jourdain[5], C. Sebrie[5], F. Pouzoulet[3,4]

* Equal contribution

1. Laboratoire d'Imagerie et Modélisation en Neurobiologie et Cancérologie (IMNC), Centre National de la Recherche Scientifique (CNRS); Universités Paris 11 and Paris 7, Campus d'Orsay, 91405 Orsay (France).
2. Histopathologie Humaine et Modèles Animaux, Institut Pasteur, 28 Rue du Docteur Roux, 75015 Paris (France)
3.Institut Curie, PSL Research University, Translational Research Department, Experimental Radiotherapy Platform, Orsay, France
4. Paris Sud University, Paris -Saclay University, 91405 Orsay, France
5. Imagerie par Résonance Magnétique Médicale et Multi-modalités (IR4M-UMR8081), Université Paris Sud, 91405 Orsay (France).

* Corresponding author: prezado@imnc.in2p3.fr

Energy spectrum of the SARPP calculated with SpeckCalc when a tension of 220 kV and an intensity of 13 mA were employed with inherent and additional filtrations of 0.8 mm and 0.15 mm of Beryllium and Copper, respectively. This results in a spectrum with an effective energy of 69 keV.

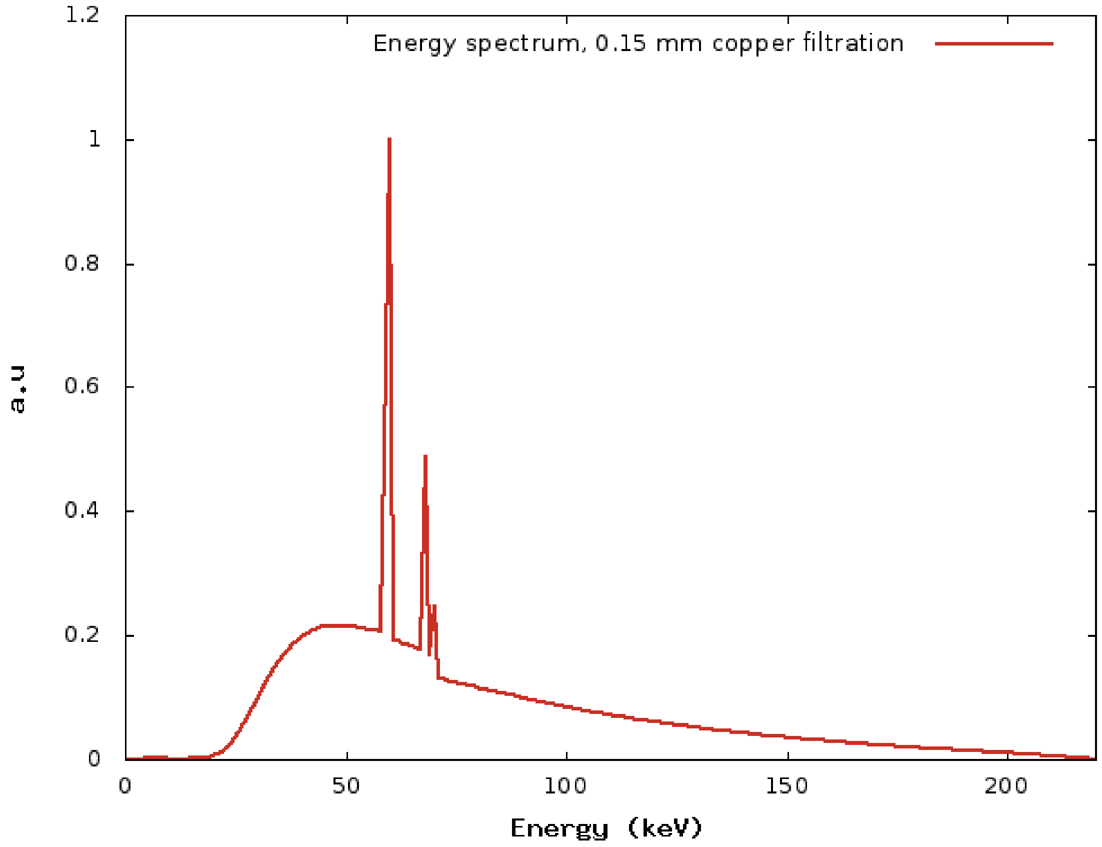

Supplement: Supplementary file 1 — Supplementary Information [file 41598_2017_17543_MOESM1_ESM.pdf]
